# Supplementary material for: Autocatalytic base editing for RNA-responsive translational control
Source: Nat Commun. 2023 Mar 11;14:1339. doi: 10.1038/s41467-023-36851-z (PMC10008589; doi:10.1038/s41467-023-36851-z)
Supplement: Supplementary file 1 — Supplementary information [file 41467_2023_36851_MOESM1_ESM.docx]

***Supplementary Information for:***

# Autocatalytic base editing for RNA-responsive translational control

Raphaël V. Gayet, Katherine Ilia, Shiva Razavi, Nathaniel D. Tippens, Makoto A. Lalwani, Kehan Zhang, Jack X. Chen, Jonathan C. Chen, Jose Vargas-Asencio, and James J. Collins

**Supplementary Figures**


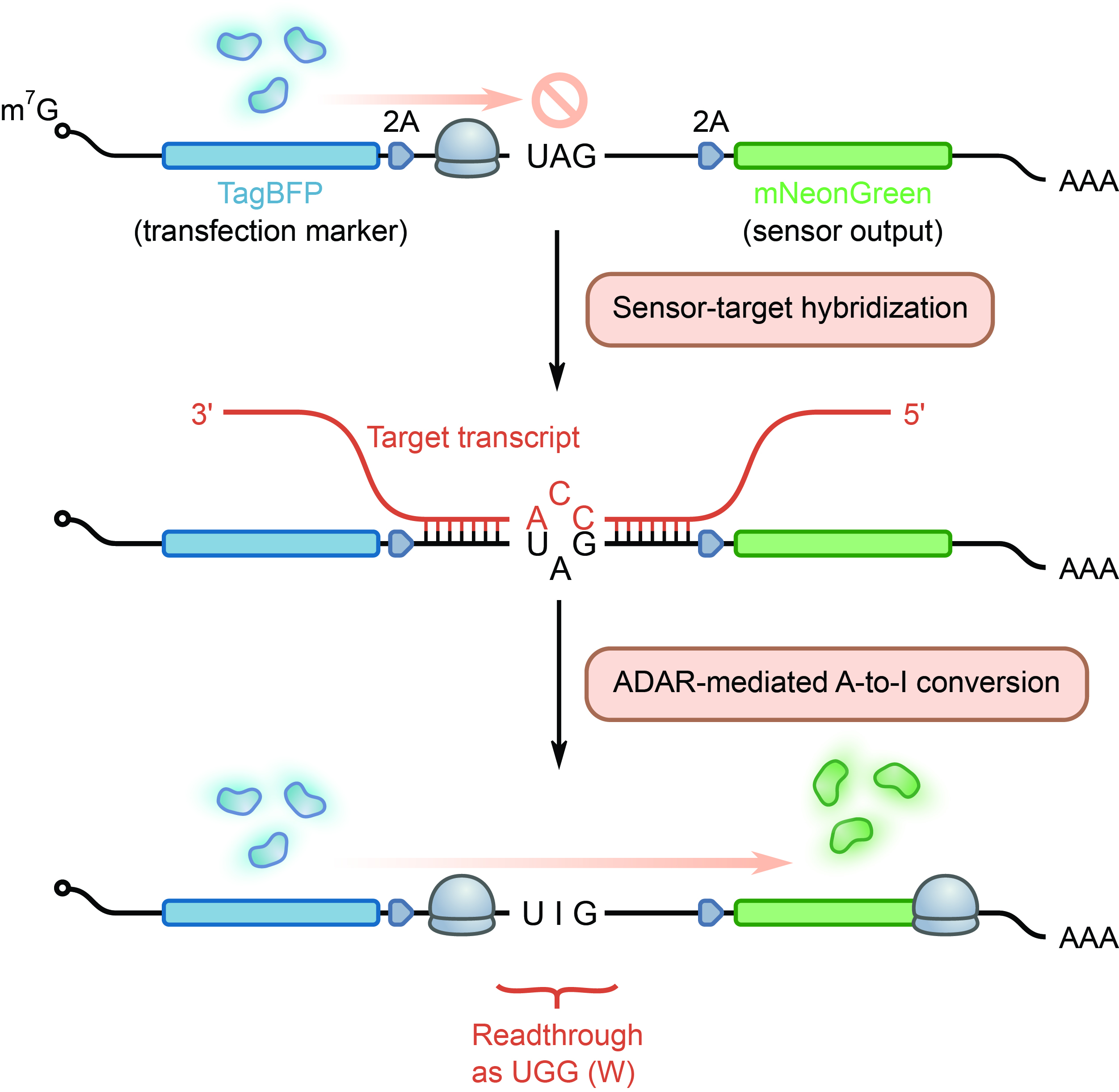


**Supplementary Figure** [**1**](#_bookmark34)**. First-generation ADAR-mediated sensor design.** First-generation ADAR-based RNA-responsive sensors, as have been previously reported, are activated by the specific hybridization of target transcripts, followed by the enzymatic deamination of the mismatched A in the central stop codon. We used this preliminary design as the basis for optimizing our DART VADAR sensors. Here, we included a TagBFP reporter sequence at the 5’ end of the sensor transcript to account for plasmid dosage, and an mNeonGreen coding sequence downstream of the sensor sequence as the output. All the elements are insulated by self-cleaving 2A peptide sequences. m7G: mRNA cap; 2A: self-cleaving 2A peptide; AAA: poly(A) tail.

**
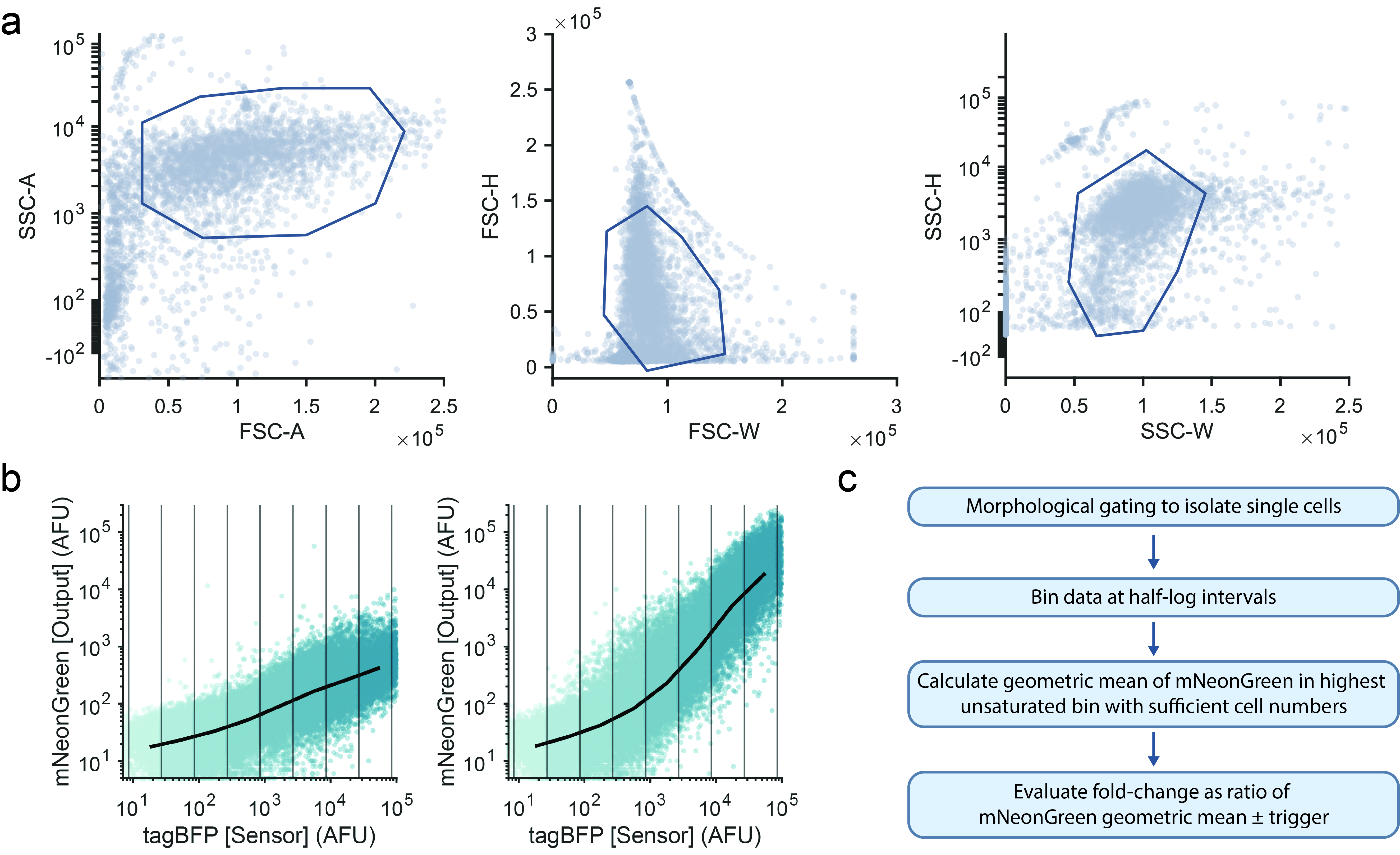
**

**Supplementary Figure** [**2**](#_bookmark35)**. Flow cytometry analysis pipeline. A.** Cells are gated based on forward- and side- scatter signals. **B.** Data acquired via flow cytometry were binned at half-log intervals, excluding datapoints with saturated fluorescence measurements. This example presents representative data for sensor CCA60 in the absence (left) or presence (right) of secreted iRFP720 trigger. **C.** The workflow presents an overview of our data processing strategy.

**
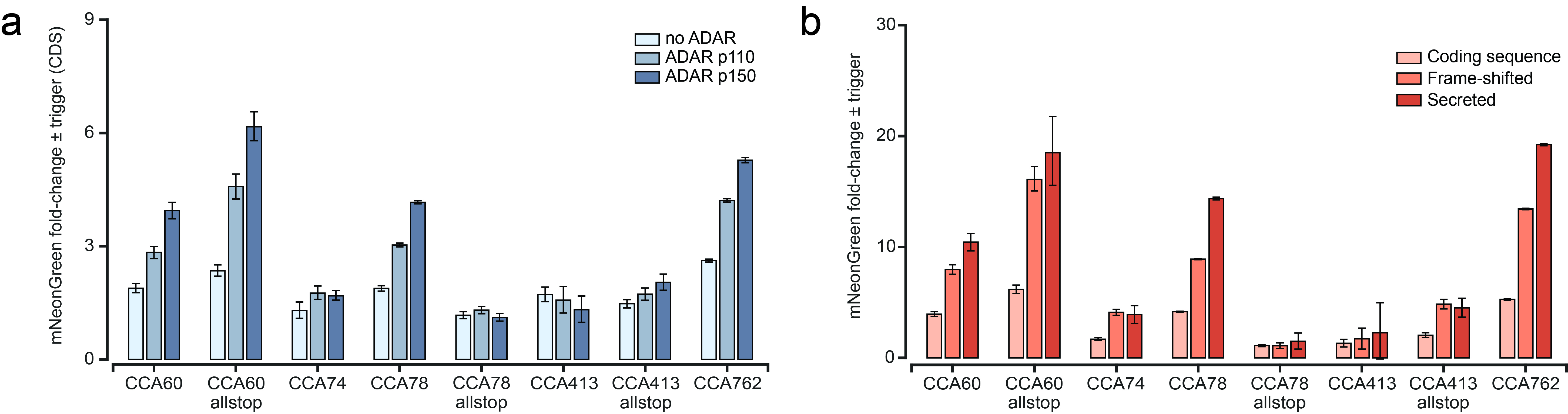
**

**Supplementary Figure** **[3](#_bookmark36). Performance of shorter ADAR-mediated sensors. A.** The exogenous supplementation of ADAR improves the performance of 51 bp sensors, although these have slightly lower dynamic range compared to the 75 bp sensors. The value of each bar corresponds to the output fold-change (FC), which is the ratio of the geometric mean of mNeonGreen expression in the presence and absence of trigger. Error bars represent 95% confidence intervals for the fold-change values, determined from at least 2000 cells. **B.** Albeit to a lesser extent than the 75 bp sensors, 51 bp ADAR-based sensors also yield higher dynamic range when targeting 3’UTRs or transcripts of secreted proteins. The value of each bar corresponds to the output fold-change (FC), which is the ratio of the geometric mean of mNeonGreen expression in the presence and absence of trigger. Error bars represent 95% confidence intervals for the fold-change values, determined from at least 2000 cells.

**
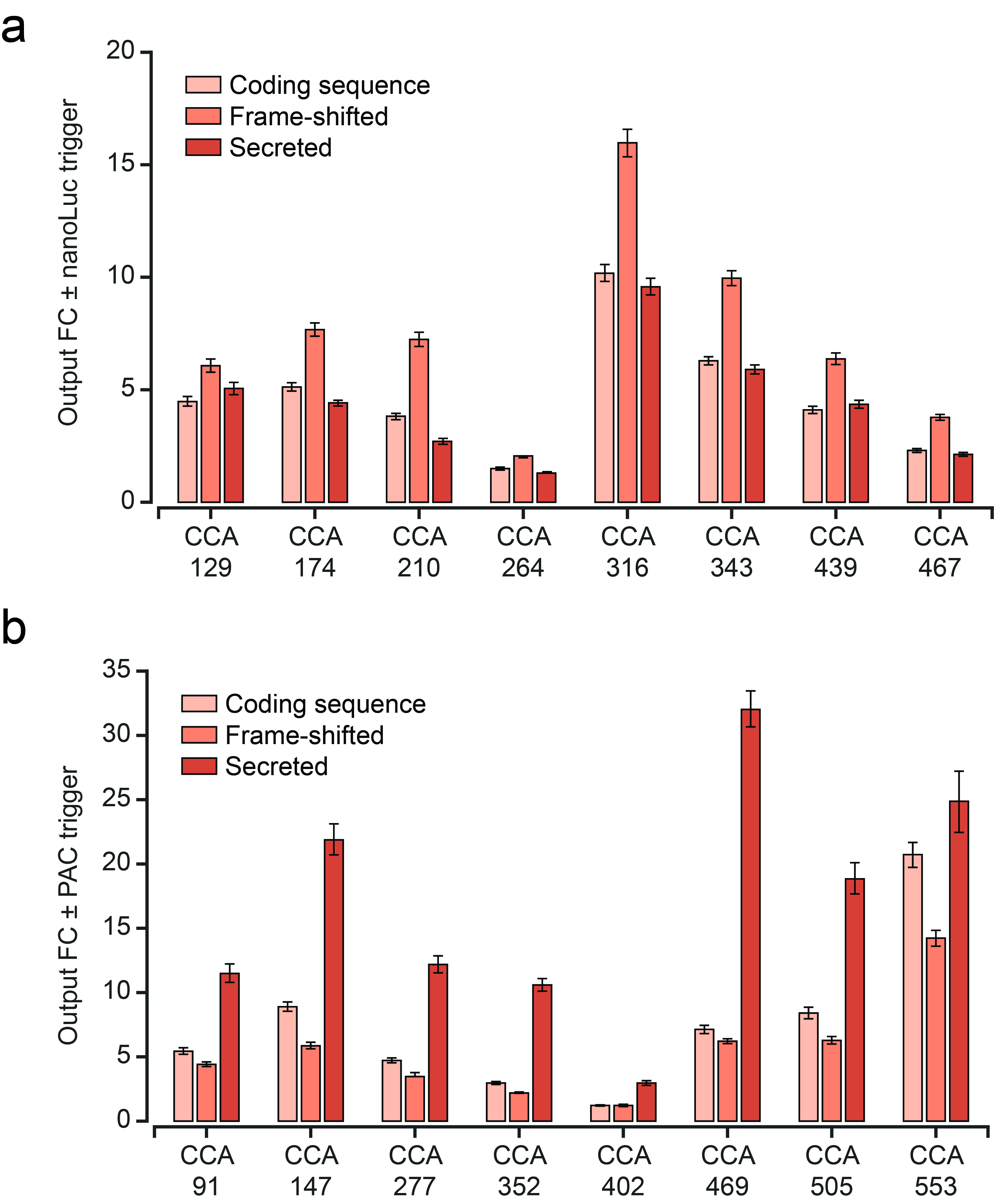
**

**Supplementary Figure** [**4**](#_bookmark36). **Performance of ADAR-based RNA sensors targeting coding sequences of cytosolic and secreted proteins, and 3’UTRs.** ADAR-based sensors yield higher dynamic range when designed to target the 3’UTRs of transcripts (as is the case for NanoLuc luciferase in panel A), or coding sequences of secreted proteins (as is the case for puromycin acetyltransferase (PAC) in panel B). The value of each bar corresponds to the output fold-change (FC), which is the ratio of the geometric mean of mNeonGreen expression in the presence and absence of trigger. Error bars represent 95% confidence intervals for the fold-change values, determined from at least 2000 cells.

**Supplementary Figure** **[5](#_bookmark37). Detecting nuclear transcripts.** ADAR-mediated RNA-responsive sensors targeting the nuclear lncRNA MALAT1, as well as exogenous ADAR p150 and p110, were transiently transfected in two A549 cell lines. WT cells: parental cell line; ∆ cells: MALAT1 knock-out derivative. The sensor output expression was comparable in both cell lines across 16 different sensors, suggesting that current ADAR-mediated RNA-responsive sensors function in the cytosol.


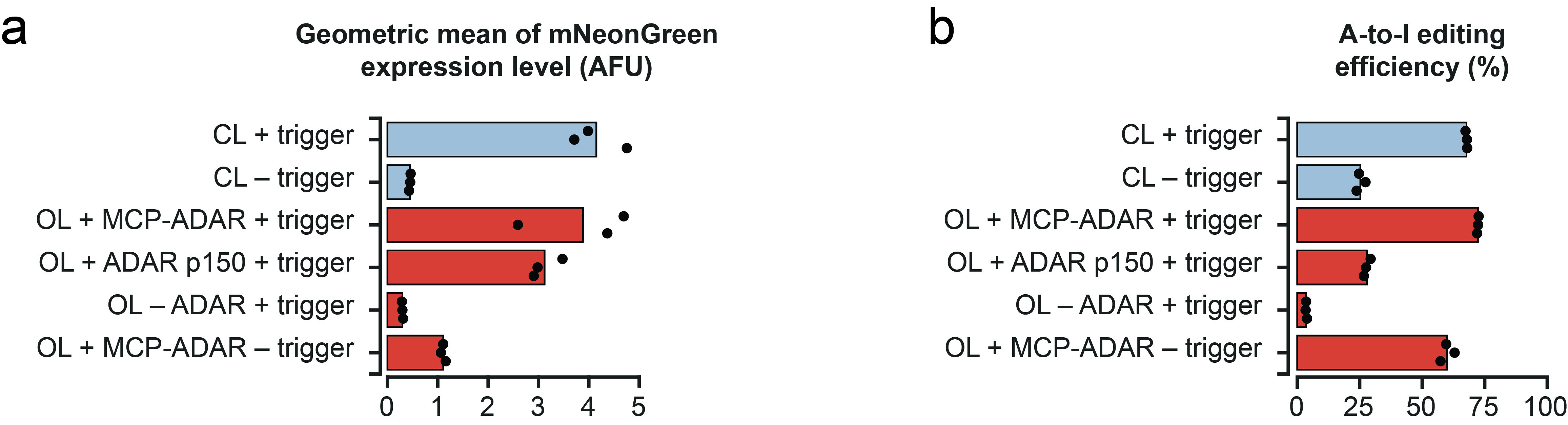


**Supplementary Figure** **[6](#_bookmark37). Changes in translational outputs reflect underlying changes in A-to-I RNA editing.** We transfected HEF293FT cells with different sensor architectures and corresponding mRNA targets. We analyzed (A) the output protein expression levels (shown in log_10_ scale) via flow cytometry and (B) the frequencies of A-to-I editing of the sensor UAG stop codon via NGS. Bars represent the mean measured on n=3 biological replicates. CL: closed-loop (DART VADAR); OL: open-loop; AFU: arbitrary fluorescent units.

**
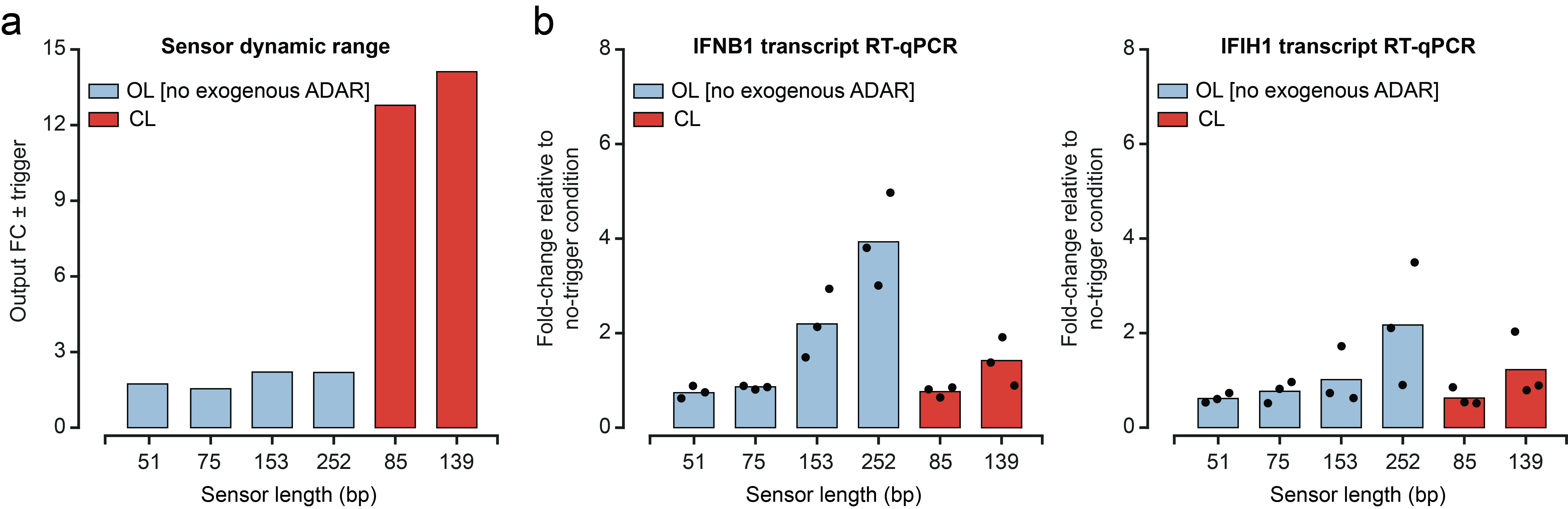
**

**Supplementary Figure** **[7](#_bookmark37). The effect of sensor length on its performance in the absence of exogenous ADAR.** (A) The performance of ADAR-based sensors relying only on endogenous ADAR is not appreciably improved with increased sensor length. FC: fold-change (B) RT-qPCR gene expression analysis highlights an increase in the expression of transcripts involved in the dsRNA immune response as a function of the length of the sensor-trigger duplexes. Fold changes represent the ratios of GAPDH-normalized transcript abundances in the presence and absence of RNA trigger. Bars represent the means measured on n=3 biological replicates. CL: closed-loop (DART VADAR); OL: open-loop.


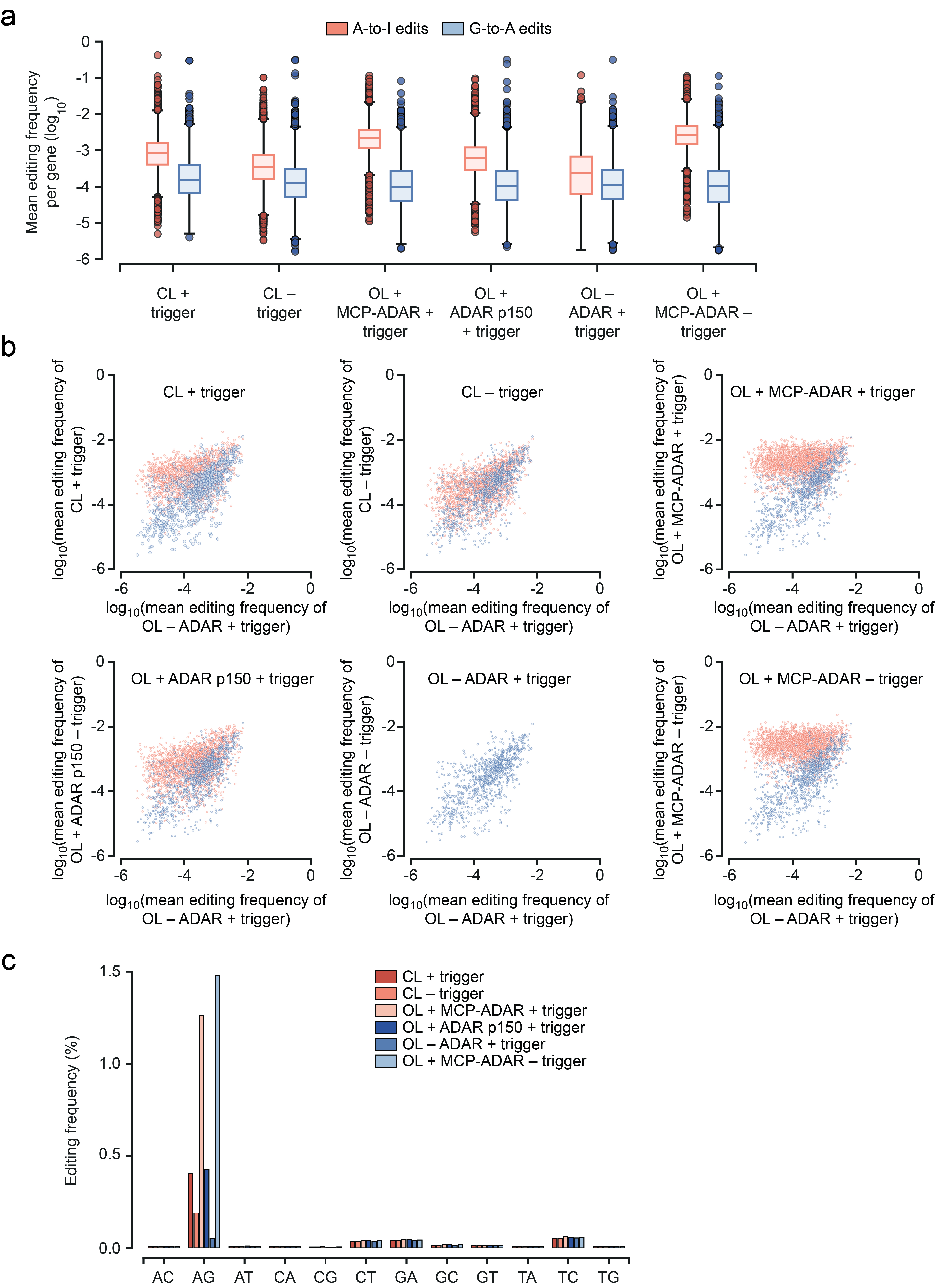


**Supplementary Figure** [**8**](#_bookmark37)**. Analysis of ADAR-mediated global off-target effects.** **a** We used RNA-seq to identify global off-target A-to-I editing in HEK293FTs transfected with various constructs. Results indicate lower off-target editing for cells expressing DART VADAR compared those expressing their open-loop counterparts. G-to-A editing is shown as a background control. Logarithmic scale omits the genes that do not exhibit measurable A-to-I editing. Box plots indicate median and interquartile ranges of all edited genes. The ends of the whiskers cover the range to the maximum and minimum data points not considered outliers, and the outliers are plotted as circles. Plots were generated using the boxplot function in Matlab, which classifies points as outliers if they are greater than q3 + w × (q3 – q1) or less than q1 – w × (q3 – q1), where w is the multiplier whisker, and q1 and q3 are the 25^th^ and 75^th^ percentiles of the sample data, respectively. **b** For each experimental condition, we plotted the mean editing frequency in log10 space against the mean editing frequency of the negative control (OL ‒ ADAR + trigger). We plotted two replicates of the OL ‒ ADAR + trigger control against each other. For ease of comparison, we overlaid this distribution (in blue) in all the scatter plots. **c** The bar graph summarizes the proportion of transcripts we identified as having the single nucleotide edits on the x-axis (e.g., AG refers to A-to-G edits).

**Supplementary Figure** [**9**](#_bookmark38)**. Guide to designing DART VADAR sensors.** We provide a step-by-step guide for designing DART VADAR sensors with MS2 hairpins.

# Supplementary Tables

# Supplementary Table 1. Oligonucleotides used in this study. We obtained the sequences for oKI_00398_f and oKI_00399_r from published reports.[^37^](#_bookmark7)

| Name | Sequence (5’ to 3’) | Application |
| --- | --- | --- |
| org1462_csnk2a2_qpcr_f | CCGGAGGCCCTAGATCTTCTTGAC | RT-qPCR for murine casein kinase 2 alpha 2 |
| org1463_csnk2a2_qpcr_r | GGGACTGCTCCTTCACCACC | RT-qPCR for murine casein kinase 2 alpha 2 |
| org1464_myog_qpcr_f | TTGCTCAGCTCCCTCAACCAG | RT-qPCR for murine myogenin |
| org1465_myog_qpcr_r | AGCCGCGAGCAAATGATCTC | RT-qPCR for murine myogenin |
| org1472_myh7_qpcr_f | GGCGCATCAAGGAGCTCACC | RT-qPCR for murine myosin heavy chain I |
| org1473_myh7_qpcr_r | CCTGCTCCTCCGCCTCCTC | RT-qPCR for murine myosin heavy chain I |
| org1476_alpl_qpcr_f | CACCTGCCTTACCAACTCTTTTGTG | RT-qPCR for murine alkaline phosphatase |
| org1477_alpl_qpcr_r | GGCTACATTGGTGTTGAGCTTTTGG | RT-qPCR for murine alkaline phosphatase |
| org1212_dv_ins_f | TTAAGGGCCTGCAGGGTG | PCR double-stranding of sensor sequences |
| org1213_dv_ins_r | TGGCTAGCCCCTCGAGT | PCR double-stranding of sensor sequences |
| CCA60-2264F | CTGAAACAGGCAGGAGATGTGGA | cDNA synthesis for NGS |
| CCA60-2463R | CCGCATGTAAGCAGACTTCCTCT | cDNA synthesis for NGS |
| org1718_qpcr_ifnb_f | TGCTCTCCTGTTGTGCTTCTCC | RT-qPCR for human IFNB1 |
| org1719_qpcr_ifnb_r | AGCCTCCCATTCAATTGCCAC | RT-qPCR for human IFNB1 |
| org1716_qpcr_mda5_f | AAGAAGAAAAAAGCATCTGAGCCTGG | RT-qPCR for human IFIH1 |
| org1716_qpcr_mda5_r | AGCCTCCCATTCAATTGCCAC | RT-qPCR for human IFIH1 |
| oKI_00398_f | ACGACCACTTTGTCAAGCTCATTTC | RT-qPCR for human GAPDH |
| oKI_00399_r | GCAGTGAGGGTCTCTCTCTTCCTCT | RT-qPCR for human GAPDH |

# Supplementary Movies

**Supplementary Movie 1:** Confluent C2C12 cells cultured in serum-restricted conditions for 7 days are capable of contracting, functionally demonstrating that differentiation towards the muscle lineage was successful.
