# Supplementary material for: Autocatalytic base editing for RNA-responsive translational control
Source: Nat Commun. 2023 Mar 11;14:1339. doi: 10.1038/s41467-023-36851-z (PMC10008589; doi:10.1038/s41467-023-36851-z)
Supplement: Supplementary file 2 — Reporting Summary [file 41467_2023_36851_MOESM2_ESM.pdf]

nature portfolio

Corresponding author(s): James J. Collins

Last updated by author(s): Feb 3, 2023

Reporting Summary

Nature Portfolio wishes to improve the reproducibility of the work that we publish. This form provides structure for consistency and transparency in reporting. For further information on Nature Portfolio policies, see our Editorial Policies and the Editorial Policy Checklist.

Statistics

For all statistical analyses, confirm that the following items are present in the figure legend, table legend, main text, or Methods section.

n/a	Confirmed	
☐	☒	The exact sample size (*n*) for each experimental group/condition, given as a discrete number and unit of measurement
☒	☐	A statement on whether measurements were taken from distinct samples or whether the same sample was measured repeatedly
☒	☐	The statistical test(s) used AND whether they are one- or two-sided<br>*Only common tests should be described solely by name; describe more complex techniques in the Methods section.*
☒	☐	A description of all covariates tested
☒	☐	A description of any assumptions or corrections, such as tests of normality and adjustment for multiple comparisons
☐	☒	A full description of the statistical parameters including central tendency (e.g. means) or other basic estimates (e.g. regression coefficient) AND variation (e.g. standard deviation) or associated estimates of uncertainty (e.g. confidence intervals)
☒	☐	For null hypothesis testing, the test statistic (e.g. *F*, *t*, *r*) with confidence intervals, effect sizes, degrees of freedom and *P* value noted<br>*Give P values as exact values whenever suitable.*
☒	☐	For Bayesian analysis, information on the choice of priors and Markov chain Monte Carlo settings
☒	☐	For hierarchical and complex designs, identification of the appropriate level for tests and full reporting of outcomes
☒	☐	Estimates of effect sizes (e.g. Cohen's *d*, Pearson's *r*), indicating how they were calculated

Our web collection on statistics for biologists contains articles on many of the points above.

Software and code

Policy information about availability of computer code

Data collection	Flow cytometry data: BD FACSDiva 6.0; RT-qPCR data: BioRad CFX Maestro 2.0; Luminescence: BMG ClarioStar 5.70 R2
Data analysis	Flow cytometry data: Matlab 2021b, using code adapted from: https://github.com/jonesr18/MATLAB_Flow_Analysis ; RT-qPCR data: BioRad CFX Maestro 2.0; luminescence: BMG MARS 3.42 R5; NGS read alignment: Geneious Prime 2022.1.1; RNA sequencing analysis: Fastp 0.23.2, STAR 2.7.10, REDItools 1.3; general data processing and plotting: Matlab 2021b and GraphPad Prism 9.2.0.

For manuscripts utilizing custom algorithms or software that are central to the research but not yet described in published literature, software must be made available to editors and reviewers. We strongly encourage code deposition in a community repository (e.g. GitHub). See the Nature Portfolio guidelines for submitting code & software for further information.

Data

Policy information about availability of data

All manuscripts must include a data availability statement. This statement should provide the following information, where applicable:

- Accession codes, unique identifiers, or web links for publicly available datasets
- A description of any restrictions on data availability
- For clinical datasets or third party data, please ensure that the statement adheres to our policy

Data availability

All data needed to evaluate the conclusions in the study can be found in the paper and/or the supplementary materials. Source data accompanying this manuscript

include measured fold-changes, editing rates, and RT-qPCR calculations. New plasmids used in this study are available for distribution from Addgene. Source data are provided with this paper. RNA sequencing data have been deposited in the Sequence Read Archive (SRA) database in the BioProject PRJNA932010 (https://www.ncbi.nlm.nih.gov/sra/?term= PRJNA932010). Raw .fcs files and other data are available from the corresponding author upon reasonable request.

Code availability
General MATLAB code for use in .fcs file processing and analysis are available under an open-source license in the GitHub repository https://github.com/jonesr18/MATLAB_Flow_Analysis. Specific .m scripts for each experiment are available from the corresponding authors upon reasonable request. The parameters used in the code for the RNA-sequencing data analysis can be found online at https://github.com/joncchen/dart_vadar.

Human research participants

Policy information about studies involving human research participants and Sex and Gender in Research.

Reporting on sex and gender	[Not applicable]
Population characteristics	[Not applicable]
Recruitment	[Not applicable]
Ethics oversight	[Not applicable]

Note that full information on the approval of the study protocol must also be provided in the manuscript.

Field-specific reporting

Please select the one below that is the best fit for your research. If you are not sure, read the appropriate sections before making your selection.

[x] Life sciences [ ] Behavioural & social sciences [ ] Ecological, evolutionary & environmental sciences

For a reference copy of the document with all sections, see nature.com/documents/nr-reporting-summary-flat.pdf

Life sciences study design

All studies must disclose on these points even when the disclosure is negative.

Sample size	Sample size for each subpopulation was imposed by the number of cells growing in culture vessels. In general, the presented summary statistic metrics were calculated on binned cell subpopulations, which contained at least 2000 cells; this number of cells ensures that the calculated metrics are not biased by noise associated with low counts. For many of the experiments, we chose a sample size of n=3 (three experimental repeats) because it is standard practice in the field.
Data exclusions	No data were excluded from the analysis, with the exception of flow cytometry data which we gated following the rationale described in the manuscript and below in this form.
Replication	We performed all key experiments at least twice; results were consistent across these replicates and the data presented in the article is representative of the trends we observed.
Randomization	[Not applicable] We performed all the experiments summarized in the manuscript using immortalized cell lines, which can reasonably be assumed to be identical when split into parallel wells for transfection.
Blinding	[Not applicable] We pre-defined metrics of success for the characterization of DART VADAR performance vs an open-loop control (i.e., fold-changes, background activation).

Reporting for specific materials, systems and methods

We require information from authors about some types of materials, experimental systems and methods used in many studies. Here, indicate whether each material, system or method listed is relevant to your study. If you are not sure if a list item applies to your research, read the appropriate section before selecting a response.

Materials & experimental systems

n/a	Involved in the study
[x]	[ ] Antibodies
[ ]	[x] Eukaryotic cell lines
[x]	[ ] Palaeontology and archaeology
[x]	[ ] Animals and other organisms
[x]	[ ] Clinical data
[x]	[ ] Dual use research of concern

Methods

n/a	Involved in the study
[x]	[ ] ChIP-seq
[ ]	[x] Flow cytometry
[x]	[ ] MRI-based neuroimaging

Eukaryotic cell lines

Policy information about cell lines and Sex and Gender in Research

Cell line source(s)	We obtained the HEK293FT cells from Invitrogen (#R70007), and the C2C12 cells from ATCC (#CRL-1772). Wild-type and MALAT1 KO A549 cells were kindly provided by Prof. Sven Diederichs (DKFZ, Germany).
Authentication	We did not authenticate the cell lines.
Mycoplasma contamination	We checked cell cultures for mycoplasma contamination using a commercial kit (Lonza MycoAlert #LT07-318); all cell lines tested negative for mycoplasma contamination.
Commonly misidentified lines (See ICLAC register)	No commonly misidentified cell lines were used in this study.

Flow Cytometry

Plots

Confirm that:

- [x] The axis labels state the marker and fluorochrome used (e.g. CD4-FITC).
- [x] The axis scales are clearly visible. Include numbers along axes only for bottom left plot of group (a 'group' is an analysis of identical markers).
- [x] All plots are contour plots with outliers or pseudocolor plots.
- [x] A numerical value for number of cells or percentage (with statistics) is provided.

Methodology

Sample preparation	We harvested cells 48 hr after transfection using trypsin-EDTA. We washed the cells three times with flow cytometry buffer, made of phosphate buffered saline without calcium or magnesium, supplemented with 1% FBS and 5 mM EDTA. We kept cells on ice until analysis or sorting.
Instrument	We collected the cytometry data on a BD LSR-II cytometer equipped with a HTS module. We performed the sorting on a BD Aria cell sorter.
Software	See "software and code" section of this form
Cell population abundance	We did not further characterize the population of transfected cells after sorting
Gating strategy	As a general strategy, we binned cell populations according to their transfection levels, at half-log intervals in the TagBFP-Pacific Blue channel (Supplementary Fig. 2).

- [x] Tick this box to confirm that a figure exemplifying the gating strategy is provided in the Supplementary Information.
