# Supplementary material for: Autocatalytic base editing for RNA-responsive translational control
Source: Nat Commun. 2023 Mar 11;14:1339. doi: 10.1038/s41467-023-36851-z (PMC10008589; doi:10.1038/s41467-023-36851-z)
Supplement: Supplementary file 5 — Description of Additional Supplementary Files [file 41467_2023_36851_MOESM5_ESM.pdf]

Title: Supplementary Movie 1:
Description: Confluent C2C12 cells cultured in serumrestricted conditions for 7 days are capable of contracting, functionally demonstrating that differentiation towards the muscle lineage was successful.
